# COVIDScholar: An automated COVID-19 research aggregation and analysis platform

**DOI:** 10.1371/journal.pone.0281147

**Published:** 2023-02-01

**Authors:** John Dagdelen, Amalie Trewartha, Haoyan Huo, Yuxing Fei, Tanjin He, Kevin Cruse, Zheren Wang, Akshay Subramanian, Benjamin Justus, Gerbrand Ceder, Kristin A. Persson

**Affiliations:** 1 Materials Sciences Division, Lawrence Berkeley National Laboratory, Berkeley, CA, United States of America; 2 Department of Materials Science & Engineering, University of California, Berkeley, Berkeley, CA, United States of America; 3 Indian Institute of Technology Roorkee, Roorkee, Uttarakhand, India; 4 Wuhan University, Wuhan, Hubei, China; 5 Molecular Foundry Division, Lawrence Berkeley National Laboratory, Berkeley, CA, United States of America; UCLA Medical School: University of California Los Angeles David Geffen School of Medicine, UNITED STATES

## Abstract

The ongoing COVID-19 pandemic produced far-reaching effects throughout society, and science is no exception. The scale, speed, and breadth of the scientific community’s COVID-19 response lead to the emergence of new research at the remarkable rate of more than 250 papers published per day. This posed a challenge for the scientific community as traditional methods of engagement with the literature were strained by the volume of new research being produced. Meanwhile, the urgency of response lead to an increasingly prominent role for preprint servers and a diffusion of relevant research through many channels simultaneously. These factors created a need for new tools to change the way scientific literature is organized and found by researchers. With this challenge in mind, we present an overview of COVIDScholar https://covidscholar.org, an automated knowledge portal which utilizes natural language processing (NLP) that was built to meet these urgent needs. The search interface for this corpus of more than 260,000 research articles, patents, and clinical trials served more than 33,000 users at an average of 2,000 monthly active users and a peak of more than 8,600 weekly active users in the summer of 2020. Additionally, we include an analysis of trends in COVID-19 research over the course of the pandemic with a particular focus on the first 10 months, which represents a unique period of rapid worldwide shift in scientific attention.

## Introduction

The scientific community responded to the COVID-19 pandemic with unprecedented speed. As a result, an enormous amount of research literature rapidly emerged, at a rate of over 250 papers a day [1]. The urgency and volume of emerging research has caused preprints to take a prominent role in lieu of traditional journals, leading to widespread usage of preprint servers for the first time in many fields, most prominently biomedical sciences [2, 3]. While this allows new research to be disseminated to the community sooner, this also circumvents the role of journals in filtering papers with flawed methodologies or unsupported conclusions and highlighting especially timely or impactful research results [4]. Additionally, the highly multi-disciplinary nature of the scientific community’s response to the pandemic lead to pertinent research being dispersed across many different preprint services and open-access journals. There was no single comprehensive repository of COVID-19 literature.

These challenges revealed the need and opportunity for new tools and methods that rethink the way in which researchers engage with the wealth of available scientific literature on rapidly evolving subjects, in particular those associated with an urgent societal need.

COVIDScholar embodies such an effort to address these issues by using natural language processing (NLP) techniques to aggregate, analyze, and search the COVID-19 research literature. We developed an automated, scalable infrastructure for scraping and integrating new research as it appears, and used it to construct a targeted corpus of over 260,000 scientific papers and documents from a broad range of disciplines. Of these, 180,000 directly concern COVID-19 and the remainder of the corpus–some 70,000 papers and research items–contains other information that may be useful to COVID-19 researchers like studies on SARS or other respiratory diseases. To make this corpus accessible and useful to the scientific community, we developed a unique literature search interface for this corpus, https://covidscholar.org, which has served over 33,000 users at an average of 2,000 monthly active users during the pandemic.

While a variety of other COVID-19 literature aggregation efforts started in response to the pandemic [5–7], COVIDScholar differs in the breadth of literature collected. In addition to the biological and medical research collected by other large-scale aggregation efforts such as CORD-19 [6] and LitCOVID [7], COVIDScholar’s collection targets the full breadth of COVID-19 research, including public health, behavioral science, physical sciences, economics, psychology, and humanities.

In this paper, we present a description of the COVIDScholar data intake pipeline, back-end infrastructure, and the NLP models used to power directed searches on the frontend search portal, which can serve as a model for future literature management efforts in new emergent situations with widespread, distributed research activities. We also present an analysis of the COVIDScholar corpus and discuss trends we observe in the dynamics of research output during the pandemic.

## Data pipeline & infrastructure

### Data collection

**Fig 1:**
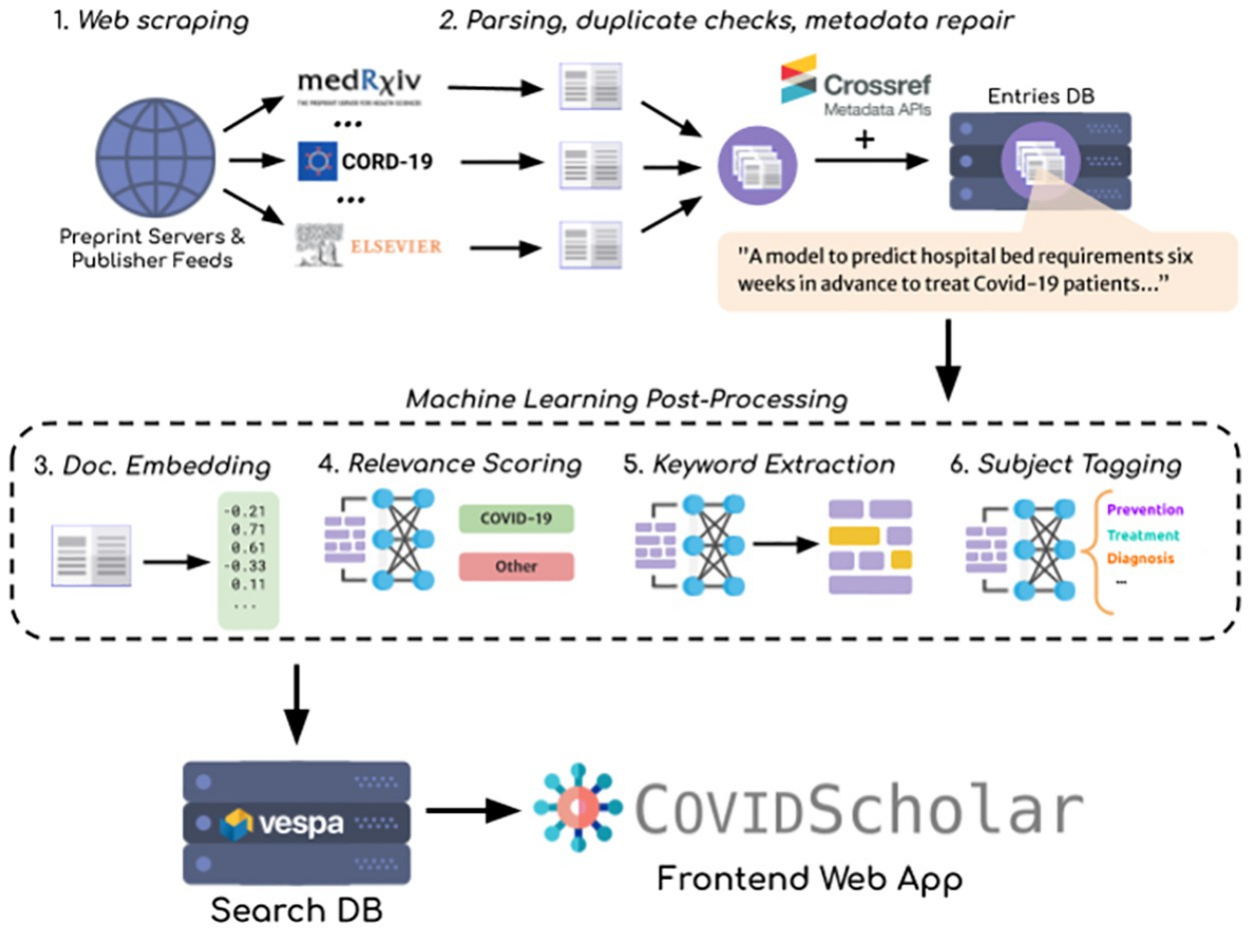
The data pipeline used to construct the COVIDScholar research corpus.

At the heart of COVIDScholar is the automated data intake and processing pipeline, depicted in Fig 1. Data sources are continually checked for new or updated papers, patents, and clinical trials. Documents are then parsed, cleaned, analyzed with NLP models to produce document embeddings, COVID-19 relevance scores, inter-document similarity metrics, keywords, and subject-area tags. The processed entries and NLP-derived metadata are then made searchable for end-users on the frontend website, https://covidscholar.org. The complete codebase for the data pipeline is available at https://github.com/COVID-19-Text-Mining.

**Fig 2:**
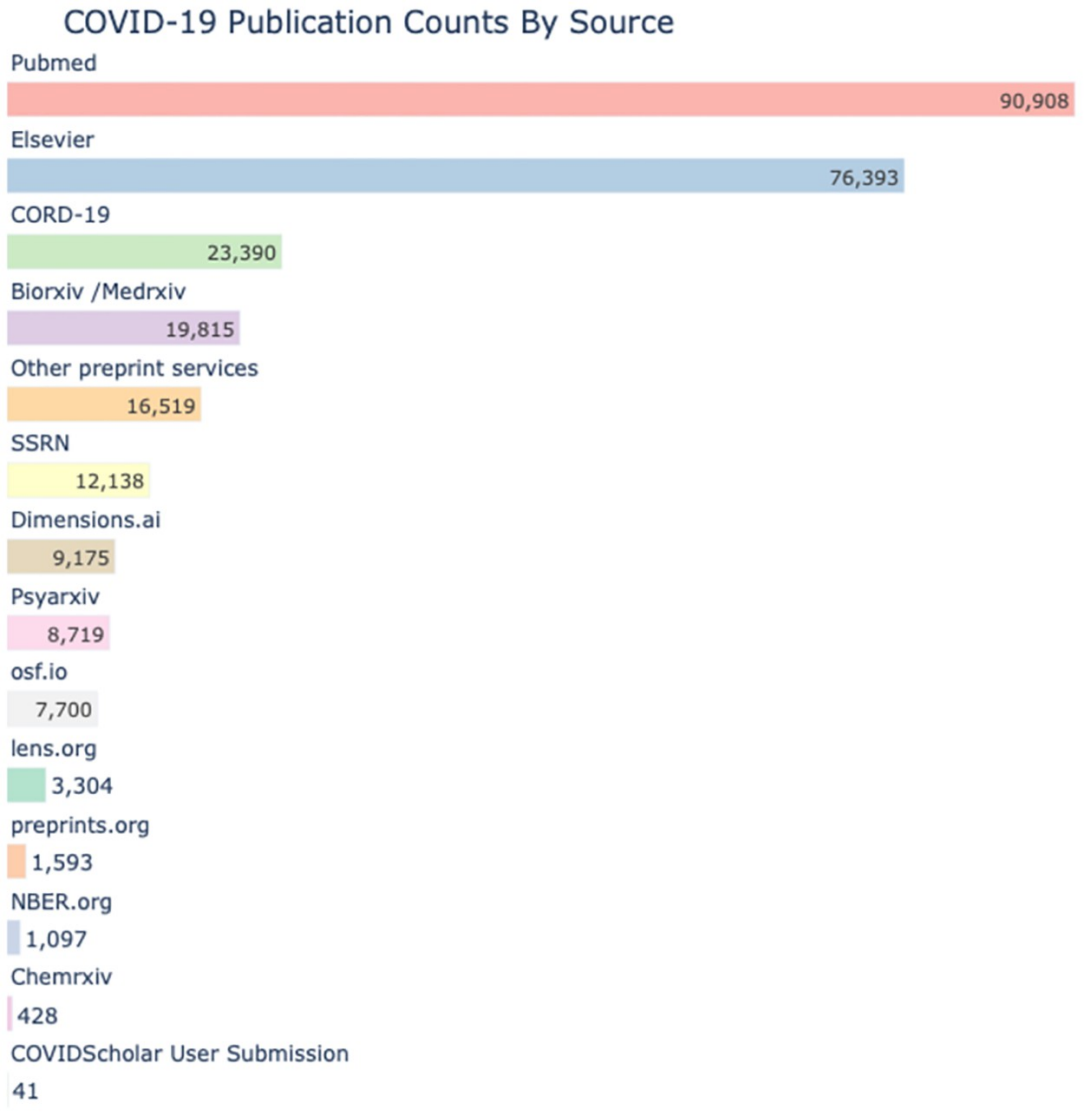
Publication counts by source. The source of papers, patents, and clinical trials in the COVIDScholar collection, with the count of COVID-19 related publications from each source. Papers are sourced from [6, 7, 9–19]. Note: many papers in our database are available from multiple sources. The total number of unique documents is approximately 260,000.

The COVIDScholar research corpus consists of research literature from 14 different open-access sources and preprint services, shown in Fig 2. For each of these, a web scraper regularly checks for new documents and new versions of existing documents. Missing metadata is then collected from Crossref and citation data is collected from OpenCitations [8]. During the early phase of the pandemic, when very little was known and response time was of the essence, the database was updated daily. Later on in the pandemic it was updated 2–3 times per week.

### Data cleaning and pre-processing

After collection, these publications are then parsed into a unified format, cleaned, and resolved to remove duplicates. Publications are identified as duplicates when they share any of doi (up to version number), pubmed id, or uncased title. For clinical trials without valid document identifiers, a shared title is used to identify duplicates. In cases where there are multiple versions of a single paper (most commonly, a preprint and a published version), a combined single document is produced, whose contents are selected on a field-by-field basis using a priority system. Published versions and higher version numbers (based on doi) are given higher priority, and sources are otherwise prioritized based on the quality of their text.

In cases where full-text PDFs are available, text is parsed from the document using pdfminer (for PDFs with embedded text [20]) or optical character recognition (OCR). However, it is our experience that text extracted in this manner is usually not of sufficient quality for to be used by the classification and relevance NLP models, and at this time is used solely for text searches.

### Overview of analysis, keyword extraction, and search index construction

We use NLP models to classify documents based on their relevance to COVID-19, topic, discipline, and field. The topic labels used are derived from the LitCovid project (https://www.ncbi.nlm.nih.gov/research/coronavirus/)—“treatment”, “prevention”, “mechanism”, “diagnosis”, “transmission”, “epidemic forecasting”, and “case report”. Documents can belong to multiple categories and publications for which an abstract cannot be found are not classified.

Keywords are also extracted from titles and abstracts using an unsupervised approach. We also apply subject/discipline labels jointly developed with the Rapid Reviews: COVID-19 editorial team for use in their back-end preprint review system—“biological & chemical sciences”, “medical sciences”, “public health”, “physical sciences and engineering”, and “humanities & social sciences”.

**Fig 3:**
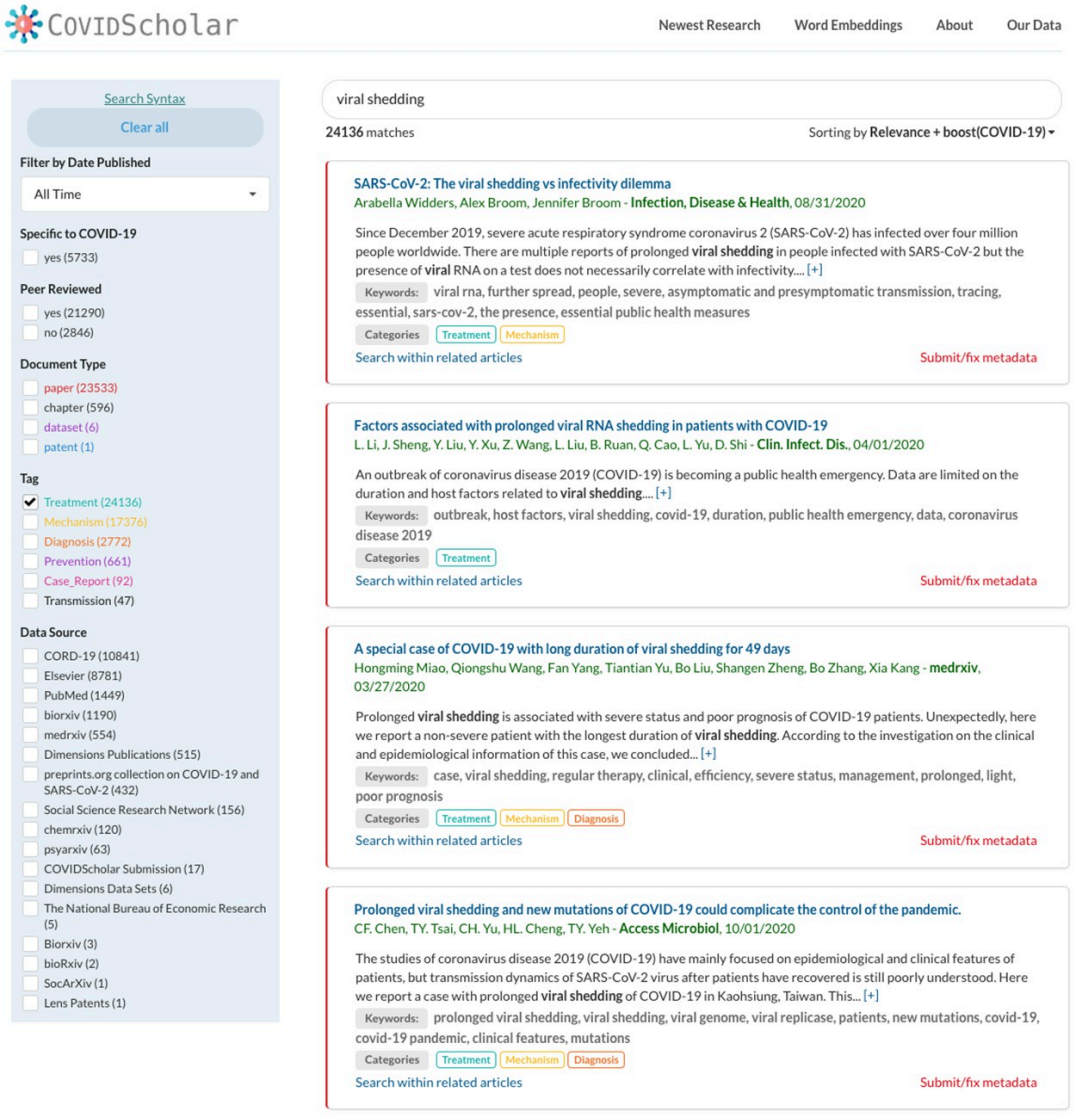
COVIDScholar literature search portal. Screenshot of COVIDScholar.org, which includes text-search with COVID-19 relevance boosting, category labels, extracted keywords, and other features designed to aid COVID-19 researchers in finding relevant information quickly and staying on top of a rapidly evolving research landscape.

Our web portal, COVIDScholar.org (Fig 3), provides an accessible user interface to a variety of literature search tools and information retrieval algorithms tuned specifically for the needs of COVID-19 researchers. To do this, we have utilized new machine learning and natural language processing techniques together with proven information retrieval approaches to create the search algorithms and indices behind COVIDScholar, which we describe in the remainder of this section.

### COVID-19 relevance scoring

The goal of any relevance scoring system is to give a higher rank to documents that are most likely to contain relevant information or answers to a user’s query. Our approach to relevance ranking in literature search and discovery for COVID-19 related research is intended to help researchers find the most relevant documents to their queries in a way that not only matches keywords in the query but also takes a document’s overall relevance to SARS-CoV2 and COVID-19 into consideration.

Machine learning algorithms can be used to identify emerging trends in the literature and correlate them with similar patterns from pre-existing research. This was especially useful in the early stages of the pandemic where little existing literature on COVID-19 had been published and researchers wanted to leverage existing research on related viruses (SARS, MERS), epidemiology, epidemiological modeling, and other respiratory diseases. For this reason, we chose to base our search backend on the Vespa engine [21], which provides a high level of performance, wide scalability, and easy integration with dense vector search and custom machine learning models. The default search result ranking profile on COVIDScholar.org combines BM25 relevance [22] (a robust keyword-based document relevance ranking algorithm) with a “COVID-19 relevance” score calculated by a classification model trained to predict whether a paper is relevant to the SARS-CoV-2 virus or COVID-19.

Ranking schemes that also promote results with information on certain viruses/diseases that are similar to SARS-CoV2 but predate the COVID-19 pandemic can be useful to COVID-19 researchers during the information discovery phase, especially for papers on the original SARS and other respiratory diseases. SARS-CoV-2 shares 79% of its genome sequence identity with the SARS-CoV virus [23], and there are many similarities between how the two viruses enter cells, replicate, and transmit between hosts [24]. Because the COVID-19 relevance classification model gives a higher score to studies on these similar diseases, we can use this score to help boost the ranking of search results that are likely to contain relevant information, even if it is not directly focused on COVID-19. A specific example of this is the transmembrane protease TMPRSS2, which plays an important role in viral entry and spread for both SARS-CoV and SARS-CoV-2. Inhibition of TMPRSS2 is a promising avenue for treating COVID-19 [25, 26]. A wealth of information on strategies to inhibit TMPRSS2 activity and their efficacy in blocking SARS-CoV from entering host cells was available in the early days of the COVID-19 pandemic and these specific studies were boosted in search results because our model assigned them high relevance scores, thereby bringing potentially useful information to the attention of researchers more directly. In comparison, results of a Google Scholar search for “TMPRSS2” (with results containing “COVID-19” and “SARS-CoV-2” filtered out) are dominated by studies on the protease’s role in various cancers rather than SARS-CoV.

COVIDScholar also provides tools that utilizes unsupervised document embeddings so that searches can be performed within “related documents” to automatically link research papers together by topics, methods, drugs, and other key pieces of information. Documents are sorted by similarity via the cosine distances between unsupervised document embeddings [27], which is then combined with the result-ranking score mentioned above. This allows users to focus their results into a more specific domain without having to repeatedly pick and choose new search terms to add to their queries. Users can also filter all of the documents in the database by broader subjects relevant to COVID-19 (treatment, transmission, case reports, etc), which are all determined though the application of machine learning models trained on a smaller number of hand-labeled examples. All combined, these approaches have allowed us to create more targeted tools for COVID-19 literature search and knowledge discovery.

## NLP models for text analysis

### Rapid reviews: COVID-19 subject model

As part of a collaboration between COVIDScholar and Rapid Reviews: COVID-19, an overlay journal that rapidly peer-reviews COVID-19 preprints, we developed a pipeline that categorized preprints into feeds from various subject areas so that editors could follow their respective areas of expertise. To do this, we trained a subject classification model on approximately 3,300 documents (title + abstract) hand-labeled with subject area tags selected by the Rapid Reviews: COVID-19 editorial team (“Biological & Chemical Sciences”, “Medical Sciences”, “Public Health”, “Physical Sciences, Engineering & Computational Studies”, and “Humanities & Social Sciences”.) A paper may belong to any number of disciplines and each discipline is composed of 12–15 sub-fields, which are listed in the S1 Table.

For this task we used a fine-tuned SciBERT [28] model trained on this datasets. While other BERT models pre-trained on scientific text exist (e.g. [29–31]), we selected SciBERT due to its broad, multidisciplinary training corpus, which we expect to more closely resemble the COVIDScholar corpus than those pre-trained on a single discipline. At the time of our model’s development, SciBERT had state-of-the-art performance on the task of paper domain classification [32], as well as a number of biomedical domain benchmarks [33–35]—the most common discipline in the COVIDScholar corpus. The input to the model is the title and abstract of the document appended together and a single fully-connected layer with sigmoid activation is used as a classification head. The model is fine-tuned for 6 epochs using roughly 3,300 human-annotated abstracts that were labeled by members of the Rapid Reviews: COVID-19 [36] editorial team. We compare this model to a baseline random forest model using TF-IDF features and two transformer text classifiers based on more recent models (distilBERT [37] and Specter [38]) trained on the same sample dataset.

## Benchmark results

**Fig 4:**
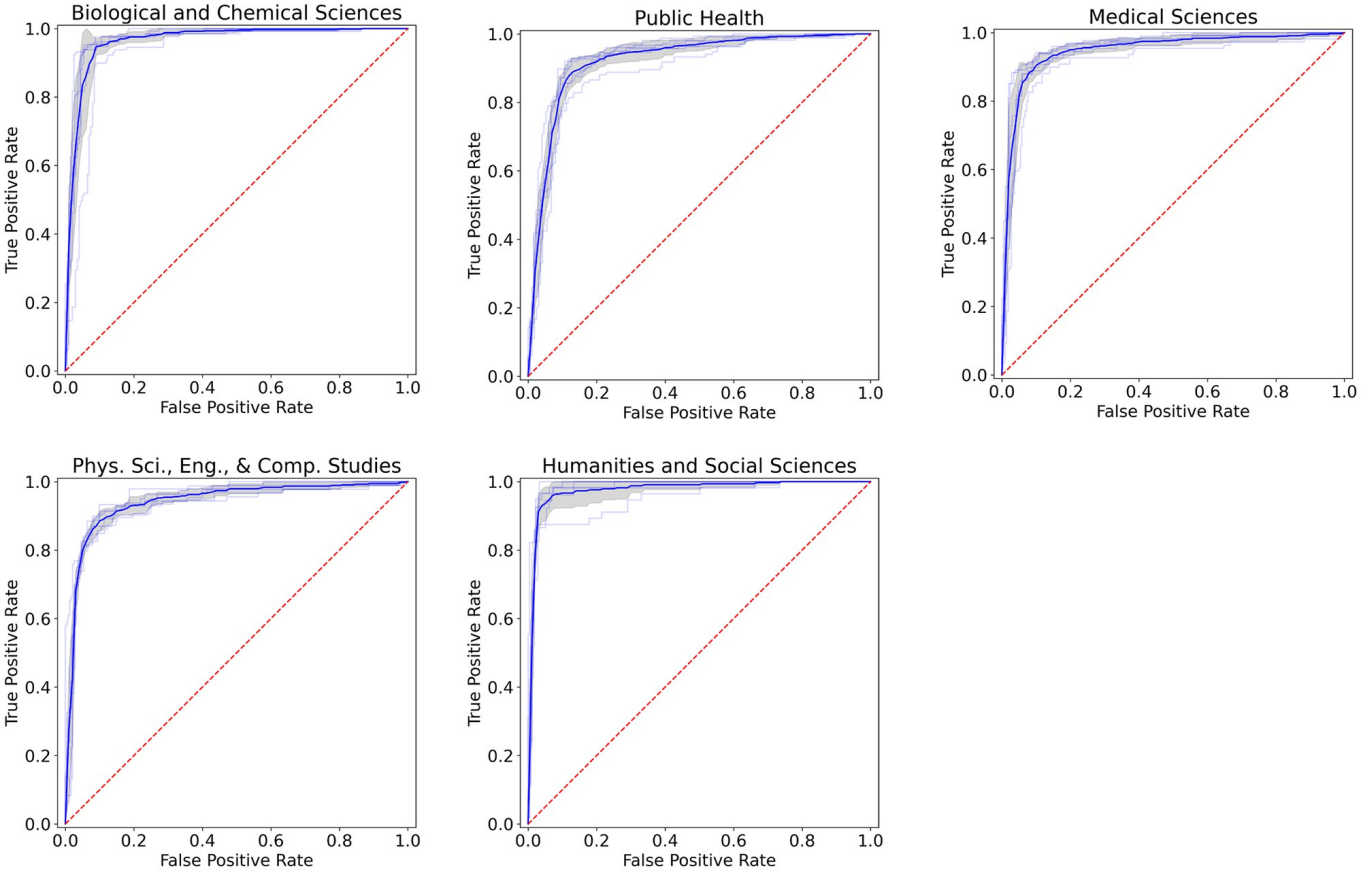
Classifier performance. ROC curves for discipline classification models of paper abstracts using a fine-tuned SciBERT [28] model adapted for classification. Training is performed using a set of roughly 3,300 human-annotated abstracts, and results shown are generated with 10-fold cross validation.

ROC curves for the COVIDScholar classifier’s performance for each top-level Rapid Reviews discipline using 10-fold cross-validation are shown in Fig 4. The classifier performs well, with average F1 scores above 0.80 for all disciplines.

Table 1 contains F1, precision, and recall scores for the Rapid Reviews category classification task for the models uses in COVIDScholar and three baseline models: a random forest model using TF-IDF features, a fine-tuned distilBERT model, and a fine-tuned Specter model. Each model was evaluated using 10-fold cross-validation over a dataset of 3,307 hand-labeled abstracts.

**Table 1:** Scoring metrics of SciBERT [28] and baseline classification models (random forest, distilBERT, and Specter).

		Biological & Chem. Sciences	Medical Sciences	Public Health	Phys. Sci, Eng. and Comp. Studies	Humanities & Social Sciences
SciBERT	F1	0.91	0.87	0.87	0.80	0.88
	Precision	0.88	0.86	0.86	0.80	0.88
	Recall	0.93	0.88	0.88	0.81	0.89
Random Forest	F1	0.79	0.51	0.68	0.26	0.46
	Precision	0.84	0.76	0.72	0.74	0.83
	Recall	0.76	0.40	0.67	0.16	0.33
distilBERT	F1	0.91	0.87	0.87	0.80	0.87
	Precision	0.84	0.86	0.84	0.79	0.88
	Recall	0.92	0.88	0.89	0.82	0.87
Specter	F1	0.90	0.87	0.86	0.81	0.87
	Precision	0.89	0.88	0.84	0.80	0.86
	Recall	0.91	0.88	0.88	0.82	0.88

The SciBERT model significantly outperforms the random forest baseline model on all categories and performs very similarly to distilBERT and Specter baseline models. It is also of note in each case that while precision is similar between the the models, the random forest baseline model exhibits significantly lower recall. This may be due to unbalanced training data—no single discipline accounts for more than 33% of the total corpus. For search applications, often a relatively small number of documents is relevant to each query. In this case, a high recall is more desirable than a high precision.

## COVID-19 relevance classification

On the task of binary classification as related to COVID-19, our current models perform similarly well, achieving an F1 score of 0.98. While the binary classification task is significantly simpler from an NLP perspective—the majority of related papers contain “COVID-19” or some synonym—this still represents a significant performance improvement over the random forest baseline model, which achieves an F1-score of 0.90. Given the relative simplicity of this task, in cases where an abstract is absent we classify it as related to COVID-19 based on the title.

## Keywords

Keywords extracted from titles and abstracts are useful for quickly summarizing search results. For the task of unsupervised keyword extraction, 63 abstracts were annotated by humans and four keyword-extraction methods were tested. Two of the methods are statistical, TextRank [39] and TF-IDF [40], and two are graph-based models, RaKUn [41] and Yake [42]. The models were evaluated for overlap between human-annotated keywords and extracted keywords, and results are shown in Table 2. Note that due to the inherent subjectivity of the keyword extraction task scores are relatively low—the best performing model, RaKUn has an F1 score of only 0.2 for recapturing the keywords chosen by the human annotator. However, the quality of extracted keywords from this model was deemed reasonable for display on the search portal after manual review.

**Table 2:** Precision, recall, and F1 scores for 4 unsupervised keywords extractors, RaKUn [41], Yake [42], TextRank [39], and TF-IDF [40]. Output from keyword extractors was compared to 63 abstracts with human-annotated keywords.

Model	Precision	Recall	F1
RaKUn	0.17	0.33	0.2
Yake	0.11	0.45	0.15
TextRank	0.06	0.36	0.09
TF-IDF	0.10	0.09	0.08

## Word and phrase embeddings

**Fig 5:**
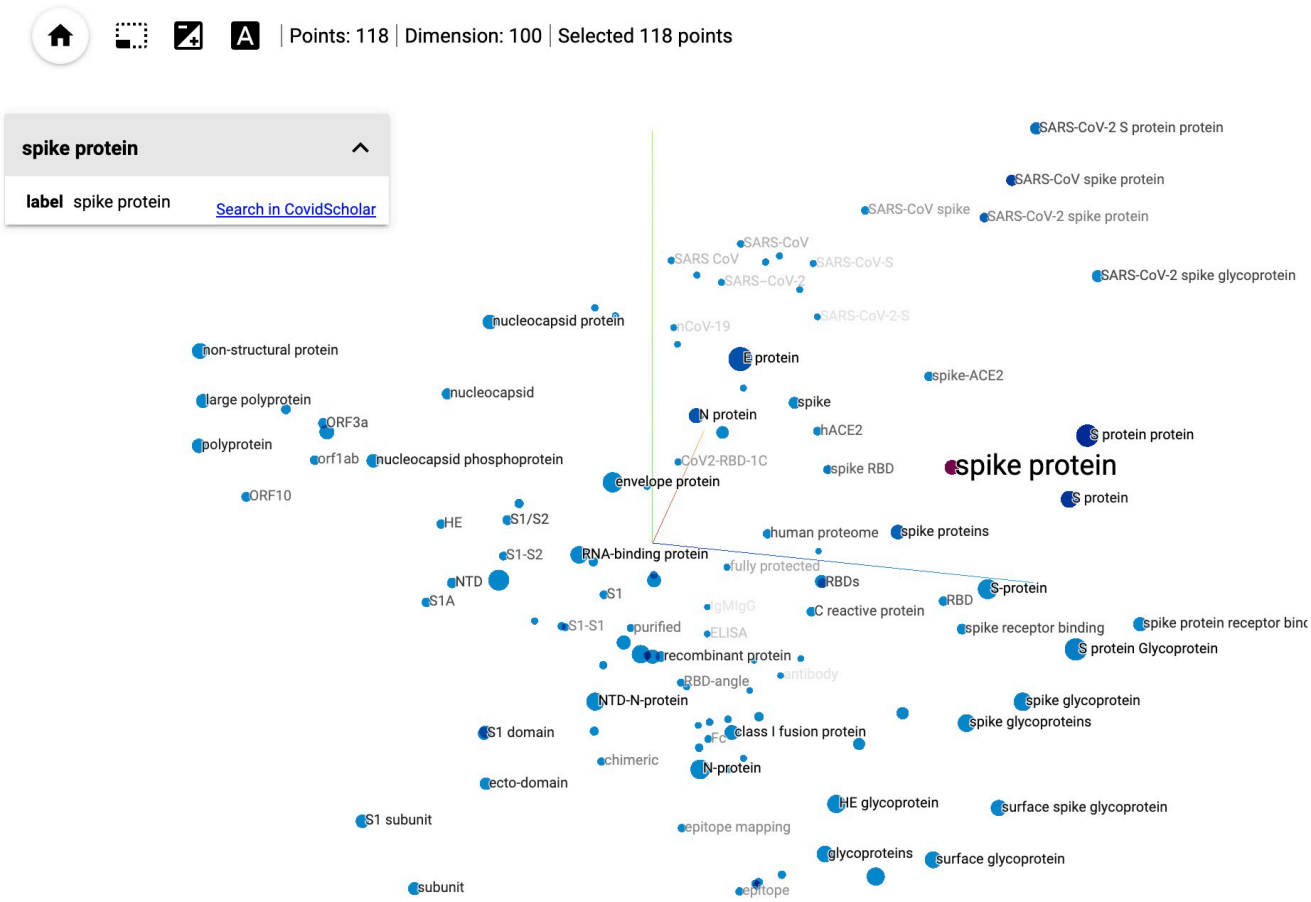
Embedding visualization. A screenshot of the embedding projector visualizing tokens similar to “spike protein”, using FastText [44] embeddings trained on the COVIDScholar corpus.

To better visualize the embedding of COVID-19-related phrases and find latent relationship between biomedical terms, we built a tool based on the work of Ref. [43] via a modified version of the open source tensorboard embedding projector visualization tool. A screenshot of the tool is shown in Fig 5. We utilize FastText [44] embeddings for the embedding projector, with an embedding dimension of 100. Embeddings are trained on the abstracts of all papers from the October 2020 COVIDScholar corpus which have been classified as relevant to COVID-19.

For the purpose of visualization, embeddings must be projected to a lower dimensional space (2D or 3D). The dimensionality reduction technique used here includes principal component analysis (PCA), uniform manifold approximation and projection (UMAP) [45] and t-distributed stochastic neighbor embedding (t-SNE) [46]. Users can set various parameters and run the dimensionality reduction algorithms in-browser, and can also load and visualize the cached result on the server with default parameters. Cosine distance is used to measure the similarity between phrases. If the cosine distance between two phrases is quite small, they are likely to have similar meaning;

$$\text{Cosine}\;\text{Distance}\left(p_{1},p_{2}\right)=1-\frac{\text{Emb}\left(p_{1}\right)\cdot \text{Emb}\left(p_{2}\right)}{∥\text{Emb}(p_{1})∥\;∥\text{Emb}(p_{2})∥}$$

where *p*_1_, *p*_2_ represent two phrases, ***Emb*** maps phrases to their embedded representation in the learned semantic space.

## COVIDScholar corpus analysis

### Corpus breakdown

As of January 2022, the COVIDScholar corpus consists of over 260,000 total documents, of which 252,000 are papers. The remainder is composed of 3,303 patents, 1,712 clinical trials, 1,194 book chapters, and 1,196 datasets. Of the papers, approximately 180,000 are classified as directly related to COVID-19. Papers marked not relevant to COVID-19 are a combination of papers on related diseases, such as SARS and MERS, or other relevant topics. In October 2020, the total number of papers was approximately equally split between preprints and published peer-reviewed papers (44% vs 56% respectively) while today the split stands at 20% preprints and 80% peer-reviewed, reflecting the evolution of the literature from a high-pace, preprint-driven modality to a more conventional mix.

To explore the subject distribution of the corpus we trained a Latent Dirichlet Allocation model on 10,000 documents (titles and abstracts) randomly selected from papers in our corpus published between Jan 2020 and Jan 2022. LDA is a probabilistic model that assumes that each document in a collection is a mixture of a fixed number of topics, and topic is a mixture of words. LDA estimates the distribution of topics in each document and the distribution of words within each topic, using a generative process [47]. We used the LDA model to assign every paper published during that time to one of 10 topics. Top keywords for each topic were used to assign a human-readable topic name, but we note that these names may not fully describe the documents belonging to each LDA topic. A table with the full lists of topic keywords is available in the S2 Table, and we also provide an interactive explorer for the LDA topic model that can be opened in a web browser at www.covidscholar.org/topics. Many of the keywords identified by our topic model were also identified by Bose et al in their analysis of COVID-19 research papers published during the pandemic [48].

**Fig 6:**
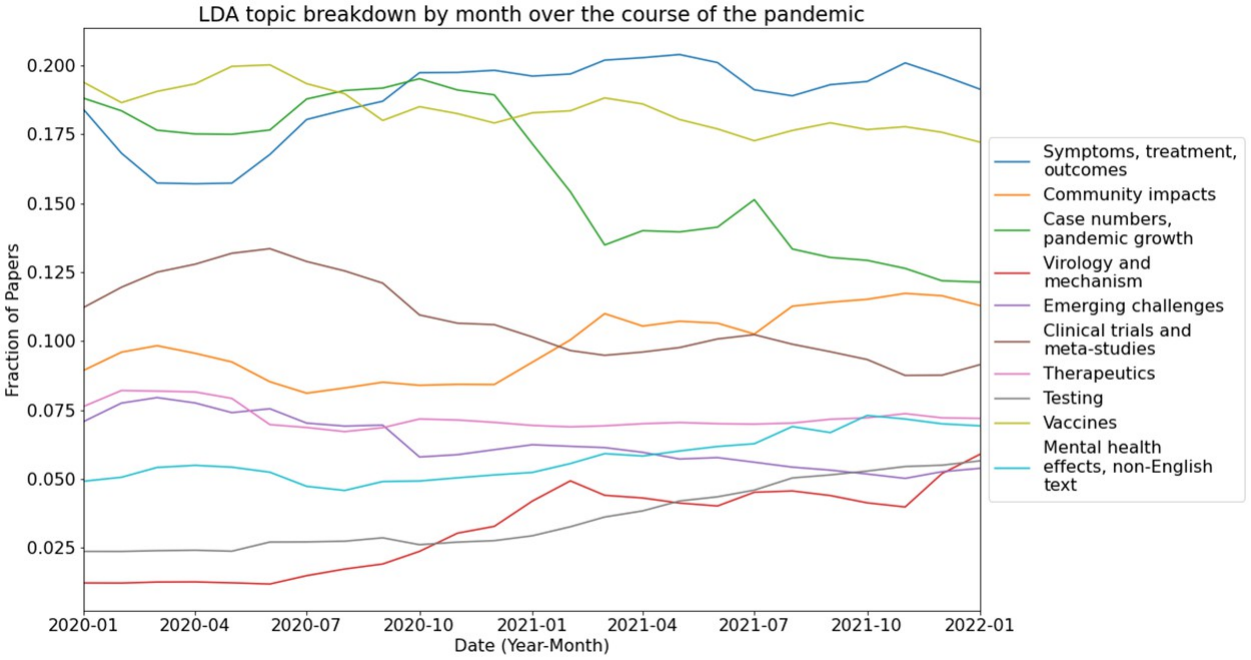
LDA topic distribution by month. Fraction of papers published each month belonging to each of the 10 LDA topics over the course of the pandemic. Lines are smoothed using the 2-month moving average.

Fig 6 shows the fraction of the papers published each month that belong to each topic over the course of the pandemic through then end of 2021 smoothed with a 2-month moving average. We observe that while most of the topics make up approximately the same fraction of papers published over the time period, the “case numbers and pandemic growth” topic shrinks significantly from approximately 18 percent of papers published per month to less than 7.5 percent while the “virology and mechanism” and “testing” topics grow from 1–2 percent to more than 5 percent. It’s interesting to note that the number of papers belonging to the testing and virology and mechanism topics make up only 10 percent of papers published in January 2022. We believe this is likely because these areas are more specialized than the other topic areas, so relatively fewer papers were published on those subjects each month.

A breakdown by discipline of the COVID-19 relevant papers sampled in October 2020 is shown in Table 3. We present cumulative counts from our database in this period as a snapshot of the research landscape as it stood in the middle of the pandemic. Approximately 85% of papers were assigned at least one category label by our model. As may be expected, “medical sciences” and “biological & chemical sciences” were the most represented disciplines, with respectively 34% and 27% of the COVIDScholar corpus at the time tagged as members of these two research areas. Overlap between these two disciplines was relatively small. Only 9,858 papers were classified as belonging to both “medical sciences” and “biological & chemical sciences” while 63,927 papers belong to only one of the two.

**Table 3:** The number of papers and fraction of total COVID-19 related papers in the COVIDScholar corpus for each discipline in October 2020, which is a good sample of the research landscape in the middle of the pandemic. Only papers with abstracts are classified and included in final count. A given paper may have any number of discipline labels or no label.

Discipline	Paper Count	Fraction of Total
Biological and Chemical Sciences	32,722	0.27
Humanities and Social Sciences	21,022	0.17
Medical Sciences	41,063	0.34
Physical Sciences, Engineering, and Computational Studies	17,413	0.14
Public Health	27,304	0.23

### Evolution of corpus size and makeup

**Fig 7:**
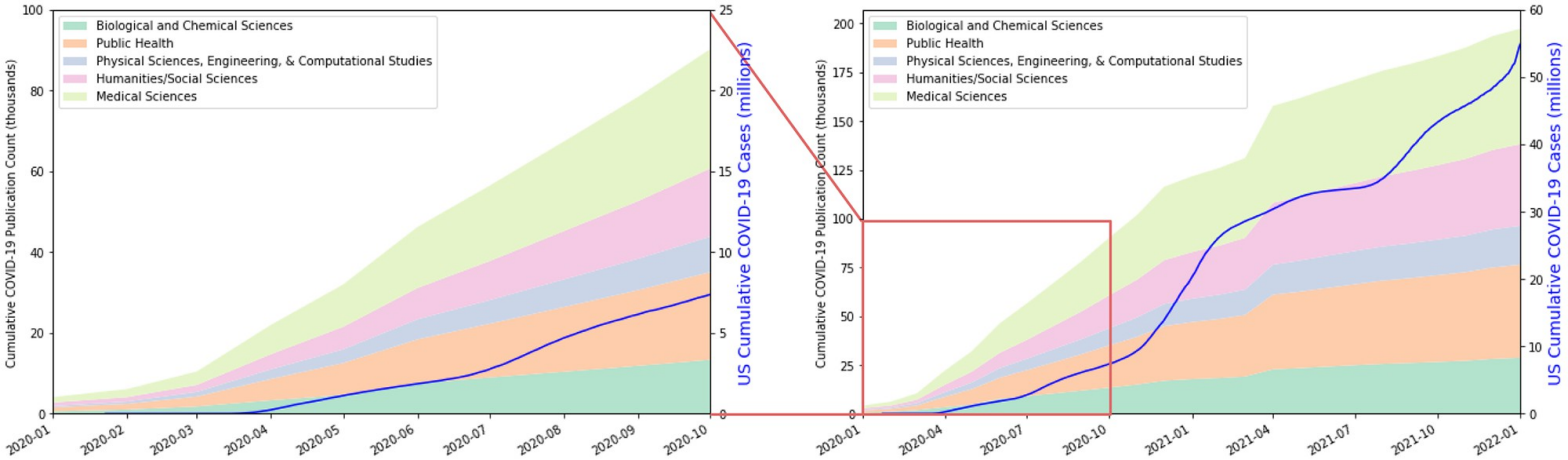
Overview of COVID-19 papers in corpus by discipline. Cumulative count by primary discipline of COVID-19 papers in the COVIDScholar database and total number of reported US COVID-19 cases from January 2020 to January 2022 (Right) and focused view of January-October 2020 (Left). Papers are categorized by the classification model described in Sec. 1. Case data from the United States Center for Disease Control. Note that only those papers with abstracts available are classified and papers can be classified with none of the labels, so the publication count is somewhat lower than the total number of documents in the database.

A cumulative monthly count of COVID-19 papers in the COVIDScholar collection over the pandemic is shown in Fig 7. Papers are categorized by the fine-tuned SciBERT model described later in “NLP models for text analysis.” In cases where a paper falls into multiple disciplines, we add it fractionally to both categories with equal weight (e.g. a paper with two categories adds 1/2 of a paper to each.) The total number of reported US COVID-19 cases is also plotted. Data on cases is from US Center for Disease Control, based on reports from state and local health agencies (https://covid.cdc.gov/covid-data-tracker/#trends_dailycases).

The rate at which publications emerged in all disciplines shows a steep increase through the early months of 2020. Between the declaration of a Public Health Emergency of International Concern [49] by the World Health Organization in January 2020 and April 2020, the rate of new publications approximately tripled every month, from just 91 papers published in January to 7,135 papers published in April. After May 2020 the rate stabilized at approximately 8,000 papers per month.

Given the lag between research activity and publication, it therefore seems that by April 2020 the COVID-19 research effort had already reached full capacity. This is before the US case count began to dramatically rise in the summer of 2020. The US government also passed two stimulus bills, each with over $1 billion in funding allocated for coronavirus research on March 5th, 2020 [50] and March 27th, 2020 [51]. Our data suggests that any increase in rate of research associated with these had already fully manifested itself within 2 months of their passing, demonstrating the rapidity of the scientific community’s COVID-19 response. Other notable events within this time frame include the declaration of global pandemic by the WHO on March 11, 2020 [52].

**Fig 8:**
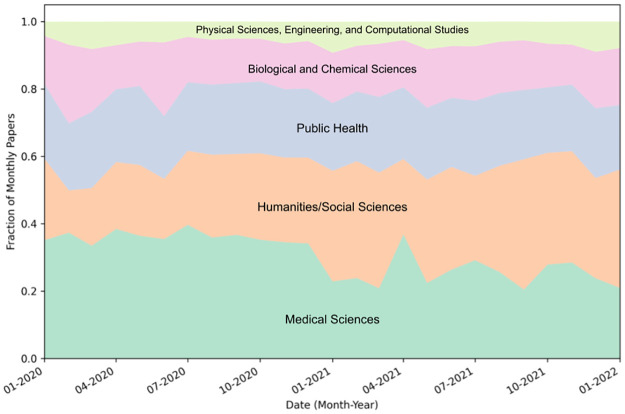
Fraction of total COVID-19 papers in corpus by primary discipline. Fractions are calculated based on total over calendar month. Papers are categorized by a fine-tuned SciBERT classification model (see “NLP models for text analysis”), and assigned to the discipline with highest predicted likelihood for this breakdown.

A breakdown of research in the COVIDScholar corpus by discipline is shown in Fig 8, which depicts the fraction of monthly COVID-19 publications primarily associated with each discipline. In this case, rather than assigning fractional papers for cases where multiple labels are assigned, we assign papers to the disciplines with the predicted label assigned the highest likelihood. There is a overall trend of an increasing fraction of research in the “humanities/social sciences” category and a decreasing fraction of research in the “medical sciences” category. The periods of February-April 2020 and June-July 2020 had more papers published in the “biological and chemical sciences” category than at other times over the pandemic, which correspond to periods where a large amount of research into the mechanism of infection and possible routes for vaccines and therapeutics was being conducted.

**Fig 9:**
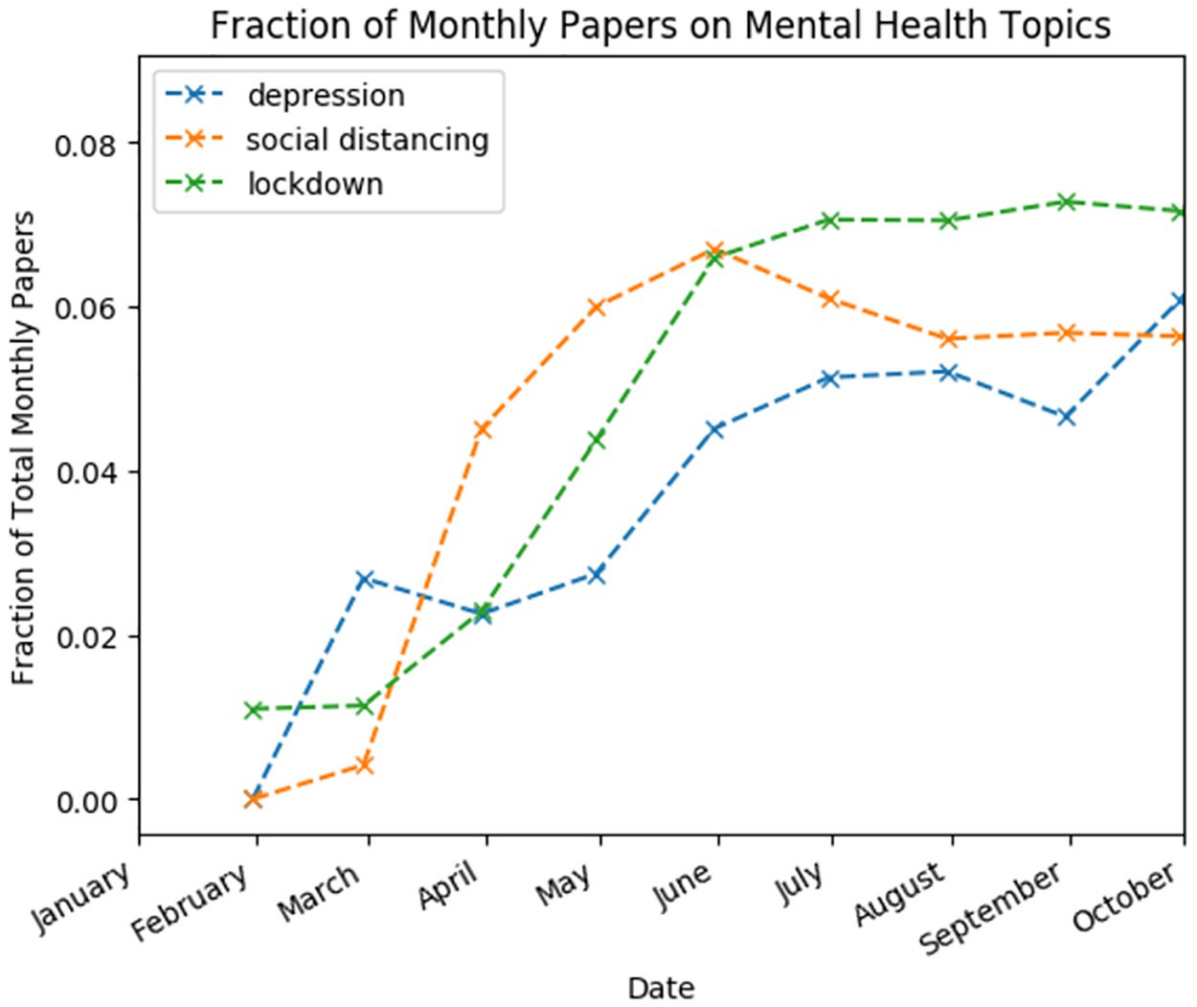
Fraction of COVID-19 literature on mental health- and lockdown-related topics on a monthly basis.

There is a clear increase in research related to psychological impacts of lockdown and social distancing. Between March and April 2020, many countries and territories instituted lockdown orders, and by April, over half of the world’s population was under either compulsory or recommended shelter-in-place orders [53]. The corresponding emergence of a robust literature on psychological impacts associated with this is the major driving force behind the increase in COVID-19 literature from “humanities & social sciences” in our corpus. This topic grew significantly during the first 10 months of the pandemic. We visualize this increase in Fig 9, where we have plotted the fraction of total monthly papers on selected topics related to mental health and lockdown.

## Summary

We developed and implemented a scalable research aggregation, analysis, and dissemination infrastructure, and created a targeted corpus of over 260,000 COVID-19 related research documents. At the height of the early pandemic, the associated search portal, https://covidscholar.org, served over 8,600 weekly scientific users at its peak and has served a total of more than 33,000 users since its release in 2020. The large amount of open data and enormous scientific interest in COVID-19 during the pandemic have made it an ideal use-case for large-scale AI-driven scientific literature aggregation efforts, but the infrastructure we have described is domain-agnostic and presents a blueprint for future situations where there is rapid production and dissemination of new research. We hope that this work and its accompanying software packages can provide useful guidance in those circumstances.

## Supporting information

S1 Table The 5 top-level disciplines (boldface) and corresponding composite fields into which COVIDScholar’s text corpus is classified. (PDF)

S2 Table LDA topics and keywords. (PDF)

## References

[1] COVIDScholar; 2020. Available from: https://covidscholar.org/stats.

[2] Johansson MA, Reich NG, Meyers LA, Lipsitch M. Preprints: An underutilized mechanism to accelerate outbreak science. PLOS Medicine. 2018;15(4):1–5. doi: 10.1371/journal.pmed.1002549 29614073 PMC5882117

[3] Fraser N, Brierley L, Dey G, Polka JK, Pálfy M, Nanni F, et al. Preprinting the COVID-19 pandemic. bioRxiv. 2020;. doi: 10.1101/2020.05.22.111294

[4] Mian A, Khan S. Coronavirus: The spread of misinformation. BMC Medicine. 2020;18(1). doi: 10.1186/s12916-020-01556-3 32188445 PMC7081539

[5] WHO COVID-19 Database; 2020. Available from: https://search.bvsalud.org/global-literature-on-novel-coronavirus-2019-ncov/.

[6] Wang LL, Lo K, Chandrasekhar Y, Reas R, Yang J, Burdick D, et al. CORD-19: The COVID-19 Open Research Dataset; 2020.

[7] Chen Q, Allot A, Lu Z. Keep up with the latest coronavirus research. Nature. 2020;579(7798):193–193. doi: 10.1038/d41586-020-00694-1 32157233

[8] Peroni S, Shotton D. OpenCitations, an infrastructure organization for open scholarship. Quantitative Science Studies. 2019;1:428–444. doi: 10.1162/qss_a_00023

[9] The Multidisciplinary Preprint Platform; 2020. Available from: https://www.preprints.org/.

[10] OSF; 2020. Available from: https://osf.io/.

[11] The Lens COVID-19 Data Initiative; 2020. Available from: https://about.lens.org/covid-19/.

[12] Social Science Research Network; 2020. Available from: https://www.ssrn.com/index.cfm/en/.

[13] Rife S. Introducing PsyArXiv: a preprint service for psychological science; 2016. Available from: http://blog.psyarxiv.com/2016/09/19/introducing-psyarxiv/.

[14] Dimensions COVID-19 Dataset; 2020. Available from: https://www.dimensions.ai/covid19/.

[15] Elsevier Novel Coronavirus Information Center; 2020. Available from: https://www.elsevier.com/connect/coronavirus-information-center.

[16] Chemrxiv; 2020. Available from: https://chemrxiv.org/.

[17] Kaiser J, Hicks L, Service RF. New Preprint Server Aims to Be Biologists’ Answer to Physicists’ arXiv; 2017. Available from: https://www.sciencemag.org/news/2013/11/new-preprint-server-aims-be-biologists-answer-physicists-arxiv.

[18] Rawlinson C, Bloom T. New preprint server for medical research; 2019. 10.1136/bmj.l2301 31167753

[19] NBER Working Papers; 2020. Available from: https://www.nber.org/papers.

[20] PDFMiner; 2020. Available from: https://github.com/pdfminer/pdfminer.six.

[21] Vespa Engine;. Available from: https://vespa.ai/.

[22] Jones KS, Walker S, Robertson SE. A probabilistic model of information retrieval: development and comparative experiments. In: Information Processing and Management; 2000. p. 779–840.

[23] Lu R, Zhao X, Li J, Niu P, Yang B, Wu H, et al. Genomic characterisation and epidemiology of 2019 novel coronavirus: implications for virus origins and receptor binding. The Lancet. 2020;395(10224):565–574. doi: 10.1016/S0140-6736(20)30251-8 32007145 PMC7159086

[24] Rabaan AA, Al-Ahmed SH, Haque S, Sah R, Tiwari R, Malik YS, et al. SARS-CoV-2, SARS-CoV, and MERS-CoV: A comparative overview. Infezioni in Medicina. 2020;28(2):174–184. 32275259

[25] Mollica V, Rizzo A, Massari F. The pivotal role of TMPRSS2 in coronavirus disease 2019 and prostate cancer. Future Oncology. 2020;16(27):2029–2033. doi: 10.2217/fon-2020-0571 32658591 PMC7359420

[26] Stopsack KH, Mucci LA, Antonarakis ES, Nelson PS, Kantoff PW. TMPRSS2 and COVID-19: Serendipity or Opportunity for Intervention? Cancer discovery. 2020;10(6):779–782. doi: 10.1158/2159-8290.CD-20-0451 32276929 PMC7437472

[27] Le Q, Mikolov T. Distributed Representations of Sentences and Documents. In: Proceedings of the 31st International Conference on International Conference on Machine Learning—Volume 32. ICML’14. JMLR.org; 2014. p. II–1188–II–1196.

[28] Beltagy I, Lo K, Cohan A. SciBERT: Pretrained Language Model for Scientific Text. In: EMNLP; 2019.

[29] Lee J, Yoon W, Kim S, Kim D, Kim S, So CH, et al. BioBERT: a pre-trained biomedical language representation model for biomedical text mining. Bioinformatics. 2019;. doi: 10.1093/bioinformatics/btz682 PMC7703786 31501885

[30] Rasmy L, Xiang Y, Xie Z, Tao C, Zhi D. Med-BERT: pre-trained contextualized embeddings on large-scale structured electronic health records for disease prediction; 2020. 10.1038/s41746-021-00455-y PMC8137882 34017034

[31] Alsentzer E, Murphy J, Boag W, Weng WH, Jin D, Naumann T, et al. Publicly Available Clinical BERT Embeddings. In: Proceedings of the 2nd Clinical Natural Language Processing Workshop. Minneapolis, Minnesota, USA: Association for Computational Linguistics; 2019. p. 72–78. Available from: https://www.aclweb.org/anthology/W19-1909.

[32] Sinha A, Shen Z, Song Y, Ma H, Eide D, Wang K. An Overview of Microsoft Academic Service (MAS) and Applications. In: WWW—World Wide Web Consortium (W3C); 2015.Available from: https://www.microsoft.com/en-us/research/publication/an-overview-of-microsoft-academic-service-mas-and-applications-2/.

[33] Yoon W, So CH, Lee J, Kang J. CollaboNet: collaboration of deep neural networks for biomedical named entity recognition. BMC Bioinformatics. 2019;20(S10). doi: 10.1186/s12859-019-2813-6 31138109 PMC6538547

[34] Nye B, Li JJ, Patel R, Yang Y, Marshall I, Nenkova A, et al. A Corpus with Multi-Level Annotations of Patients, Interventions and Outcomes to Support Language Processing for Medical Literature. In: Proceedings of the 56th Annual Meeting of the Association for Computational Linguistics (Volume 1: Long Papers). Melbourne, Australia: Association for Computational Linguistics; 2018. p. 197–207. Available from: https://www.aclweb.org/anthology/P18-1019. PMC6174533 30305770

[35] Lim S, Kang J. Chemical–gene relation extraction using recursive neural network. Database. 2018;2018. doi: 10.1093/database/bay060 29961818 PMC6014134

[36] Rapid Reviews: COVID-19, publishes reviews of COVID-19 preprints. Rapid Reviews COVID-19. 2020;.

[37] Sanh V, Debut L, Chaumond J, Wolf T. DistilBERT, a distilled version of BERT: smaller, faster, cheaper and lighter. ArXiv. 2019;.

[38] Cohan A, Feldman S, Beltagy I, Downey D, Weld DS. SPECTER: Document-level Representation Learning using Citation-informed Transformers. ArXiv. 2020;.

[39] Mihalcea R, Tarau P. TextRank: Bringing Order into Text. In: Proceedings of the 2004 Conference on Empirical Methods in Natural Language Processing. Barcelona, Spain: Association for Computational Linguistics; 2004. p. 404–411. Available from: https://www.aclweb.org/anthology/W04-3252.

[40] Salton G, Buckley C. Term-weighting approaches in automatic text retrieval. Information Processing & Management. 1988;24(5):513–523. doi: 10.1016/0306-4573(88)90021-0

[41] Skrlj B, Repar A, Pollak S. RaKUn: Rank-based Keyword extraction via Unsupervised learning and Meta vertex aggregation. ArXiv. 2019;abs/1907.06458.

[42] Campos R, Mangaravite V, Pasquali A, Jorge A, Nunes C, Jatowt A. YAKE! Collection-Independent Automatic Keyword Extractor; 2018.

[43] Smilkov D, Thorat N, Nicholson C, Reif E, Viégas FB, Wattenberg M. Embedding projector: Interactive visualization and interpretation of embeddings. arXiv preprint arXiv:161105469. 2016;.

[44] Bojanowski P, Grave E, Joulin A, Mikolov T. Enriching Word Vectors with Subword Information. arXiv preprint arXiv:160704606. 2016;.

[45] McInnes L, Healy J, Melville J. UMAP: Uniform Manifold Approximation and Projection for Dimension Reduction; 2018. Available from: http://arxiv.org/abs/1802.03426.

[46] van der Maaten L, Hinton G. Visualizing Data using t-SNE. Journal of Machine Learning Research. 2008;9:2579–2605.

[47] Blei DM, Ng AY, Edu JB. Latent Dirichlet Allocation Michael I. Jordan; 2003.

[48] Bose P, Roy S, Ghosh P. A Comparative NLP-Based Study on theCurrent Trends and Future Directions in COVID-19 Research. IEEE Access. 2021;9. doi: 10.1109/ACCESS.2021.3082108 34786315 PMC8545210

[49] WHO. Statement on the second meeting of the International Health Regulations (2005) Emergency Committee regarding the outbreak of novel coronavirus (2019-nCoV); 2020. Available from: http://bit.ly/3J7QbNI.

[50] 116th Congress (2019-2020). H.R.6074—Coronavirus Preparedness and Response Supplemental Appropriations Act, 2020; 2020. Available from: https://www.congress.gov/bill/116th-congress/house-bill/6074/text.

[51] 116th Congress (2019-2020). H.R. 748—Coronavirus Aid, Relief, 3 and Economic Security Act; 2020. Available from: https://www.congress.gov/116/bills/hr748/BILLS-116hr748eas.pdf.

[52] WHO. WHO Director-General’s opening remarks at the media briefing on COVID-19; 2020. Available from: http://bit.ly/3kxGDRX.

[53] Sandford A. Coronavirus: Half of humanity now on lockdown as 90 countries call for confinement; 2020. Available from: http://bit.ly/404odZo.

