# Supplementary material for: COVIDScholar: An automated COVID-19 research aggregation and analysis platform
Source: PLoS One. 2023 Feb 1;18(2):e0281147. doi: 10.1371/journal.pone.0281147 (PMC9891495; doi:10.1371/journal.pone.0281147)
Supplement: S1 Table — (PDF) [file pone.0281147.s001.pdf]

| **Biological & Chemical Sciences** | **Medical Sciences** | **Public Health** | **Physical Sciences** | **Humanities/Social Sciences** |
|---|---|---|---|---|
| Virology | Pathophysiology | Epidemiology | Engineering | Education |
| Immunology | Genetics | Biostatistics/ Disease Modelling | Physics | Psychology |
| Genetics/ Genomics/ Epigenetics | Clinical Management | Health Policy | Data Science | Anthropology |
| Biomedical Engineering/ Biotechnology | Infectious Disease | Nutrition/ Food Science | Mathematics Journalism and Communications | Agriculture & Resource Management |
| Vaccinology | Rheumatology | Community Health | Computational Sciences | Law & Ethics |
| Toxicology | Pulmonology/ Critical Care | Environmental Health | Materials Science | Economics |
| Molecular & Cell Biology | Geriatrics | Occupational Health | Chemistry & Chemical Engineering | Political Science |
| Integrative Biology | Emergency Medicine | Implementation Science | Statistics | International Relations |
| Ecology, Evolution, Biodiversity | Cardiology | Health Diplomacy | Climate Science | Design/ Arts/ Literature/ Music |
| Plant Biology | Pediatrics | Health Communications | Earth Sciences/ Geosciences | Sociology |
| Biochemistry | Obstetrics & Gynecology | Behavioural Health | Zoology | Business |
| Pathology/ Laboratory Medicine | Nephrology | Non-Communicable Diseases | Botany | Gender, Sexuality & Women's Studies |
| Zoonotic Diseases | Clinical Trials | | | History |
| | Primary Care | | | Area Studies |
| | Pharmacology | | | Philosophy |

**S1 Table:** The 5 top-level disciplines (boldface) and corresponding composite fields into which COVIDScholar's text corpus is classified.
