# Supplementary material for: COVIDScholar: An automated COVID-19 research aggregation and analysis platform
Source: PLoS One. 2023 Feb 1;18(2):e0281147. doi: 10.1371/journal.pone.0281147 (PMC9891495; doi:10.1371/journal.pone.0281147)
Supplement: S2 Table — (PDF) [file pone.0281147.s002.pdf]

Topic number	Assigned Topic Name	Keywords
1	Symptoms, treatment, outcomes	patient, covid, disease, hospital, severe, clinical, study, case, high, risk, symptom, coronavirus, infection, mortality, associate, care, day, group, acute, outcome, treatment, respiratory, age, include, result, year, report, conclusion, pneumonia, admission
2	Community impacts	pandemic, health, covid, care, use, study, experience, social, survey, healthcare, student, practice, work, support, impact, participant, child, result, need, online, face, medical, service, response, challenge, self, provide, change, community, include
3	Transmission and modeling	covid, pandemic, case, health, country, population, datum, rate, number, disease, outbreak, lockdown, measure, impact, risk, use, time, increase, epidemic, public, high, study, spread, result, model, period, state, death, infection, coronavirus
4	Virology and mechanism	cov, sar, infection, virus, coronavirus, covid, respiratory, disease, viral, syndrome, severe, ace, acute, cause, human, cell, variant, immune, transmission, novel, gene, pandemic, influenza, vaccine, expression, ma, sample, sequence, clinical, receptor
5	Emerging challenges	research, study, effect, human, analysis, use, development, approach, social, role, paper, suggest, process, different, provide, change, new, present, information, theory, discuss, model, base, network, understand, relationship, individual, science, aniotesin, medium
6	Clinical trials and meta-studies	study, use, covid, trial, treatment, clinical, review, analysis, effect, control, research, group, outcome, result, risk, datum, evicence, include, report, method, participant, search, intervention, base, systematic, safety, efficacy, meta, increase, protocol
7	Therapeutics	protein, drug, use, cell, virus, target, bind, antiviral, viral, inhibitor, treatment, potential, activity, therapeutic, study, molecular, compound, interaction, ma, human, structure, anti, covid, base, host, protease, replication, effective, effect, complex
8	Testing	model, test, use, method, performance, result, covid, base, testing, detection, datum, sensitivity, sample, pcr, time, positive, diagnostic, prediction, purpose, predict, accuracy, learning, image, value, parameter, une, algorithm, pregnant, machine, simulation
9	Vaccines	vaccine, antibody, cell, vaccination, cov, sar, response, assay, air, dose, level, immune, anti, plasma, antigen, blood, igg, immunity, serum, concentration, specific, spike, high, rbd, sample, low, induce, neutralizing, individual, result
10	Mental health effects, non-English text	anxiety, menal, dan, stress, depression, psychological, symptom dalam, hcw, yang, dengan, untuk, disorder, distress, health, sleep, worker, obat, covid, ini, dari, adalah, trauma, associate, pada, factor, scale, fear, sebagai, depressive

S2 Table: LDA Topics and Keywords
